# Reproducibility of Lesion Count in Various Subregions on MRI Scans in Multiple Sclerosis

**DOI:** 10.3389/fneur.2022.843377

**Published:** 2022-05-10

**Authors:** Bence Bozsik, Eszter Tóth, Ilona Polyák, Fanni Kerekes, Nikoletta Szabó, Krisztina Bencsik, Péter Klivényi, Zsigmond Tamás Kincses

**Affiliations:** ^1^Department of Neurology, University of Szeged, Szeged, Hungary; ^2^Department of Radiology, University of Szeged, Szeged, Hungary

**Keywords:** multiple sclerosis, lesion, MRI, intraclass correlation, interobservator variability, reproducibility

## Abstract

**Purpose:**

Lesion number and burden can predict the long-term outcome of multiple sclerosis, while the localization of the lesions is also a good predictive marker of disease progression. These biomarkers are used in studies and in clinical practice, but the reproducibility of lesion count is not well-known.

**Methods:**

In total, five raters evaluated T2 hyperintense lesions in 140 patients with multiple sclerosis in six localizations: periventricular, juxtacortical, deep white matter, infratentorial, spinal cord, and optic nerve. Black holes on T1-weighted images and brain atrophy were subjectively measured on a binary scale. Reproducibility was measured using the intraclass correlation coefficient (ICC). ICCs were also calculated for the four most accurate raters to see how one outlier can influence the results.

**Results:**

Overall, moderate reproducibility (ICC 0.5–0.75) was shown, which did not improve considerably when the most divergent rater was excluded. The areas that produced the worst results were the optic nerve region (ICC: 0.118) and atrophy judgment (ICC: 0.364). Comparing high- and low-lesion burdens in each region revealed that the ICC is higher when the lesion count is in the mid-range. In the periventricular and deep white matter area, where lesions are common, higher ICC was found in patients who had a lower lesion count. On the other hand, juxtacortical lesions and black holes that are less common showed higher ICC when the subjects had more lesions. This difference was significant in the juxtacortical region when the most accurate raters compared patients with low (ICC: 0.406 CI: 0.273–0.546) and high (0.702 CI: 0.603–0.785) lesion loads.

**Conclusion:**

Lesion classification showed high variability by location and overall moderate reproducibility. The excellent range was not achieved, owing to the fact that some areas showed poor performance. Hence, putting effort toward the development of artificial intelligence for the evaluation of lesion burden should be considered.

## Introduction

Multiple sclerosis (MS) is a chronic demyelinating autoimmune disease of the central nervous system. MRI has an important role in the diagnosis and follow-up of the disease. It is a sensitive tool in identifying the prominent features of the disease, lesions in the white matter, gray matter, and the spinal cord. Therefore, MRI has become the cornerstone of the diagnosis (1, 2).

The number of white matter lesions and the lesion volume have been associated with the long-term outcome of the disease (3–5). It seems that certain lesion locations have special predictive value for disease progression. The number of cortical lesions was shown to have predictive value for the clinical outcome (6, 7). Infratentorial and spinal lesions have an outstanding role in the development of clinically significant disabilities (8, 9).

Consequently, it is not only important to estimate the number of lesions to establish the dissemination of the disease. Lesion burden and location serve as predictive factors for long-term outcomes. A high lesion load might help to characterize patients with highly active multiple sclerosis (10), a patient population that may require a different therapeutic approach.

Various bodies of literature on imaging provide sufficient information about structured reporting in multiple sclerosis, which is also recommended by the recent guideline of the MAGNIMS society. Typical features of the disease were depicted in various templates (11, 12) considered to provide adequate information for neurologists. These templates are common in estimating the number of T2 hyperintense lesions in different brain regions.

The reproducibility of the lesion counts was scarcely investigated (13–15). Most of these studies were conducted in the early times of MS imaging on low magnetic field strength. They concentrated on diagnostic performance, rather than the number of lesions in various regions.

While it is known that the manual lesion segmentation is cumbersome and it is expected that there are issues with reproducibility, these were not systematically evaluated with the current state-of-the-art images. This issue is crucially important since deep learning-based automatic lesion segmentation approaches often rely on manually segmented teaching data.

In our study, we aimed to (1) determine the lesion detection disparity of five raters in the clinically relevant lesion localizations; (2) the impact of expertise in MS radiology on the identification of lesions; (3) the effect of lesion-load on the raters' performance.

## Methods

### Participants

We gathered 140 random clinical MRI scans from our MS database who suffered from the relapsing remitting form of the disease. Diagnosis of MS was performed according to the 2010 McDonald criteria (16). A detailed clinical report was available about each patient at every 3 months. The inclusion criteria was as follows: relapsing-remitting disease course with no relapse 6 months before or after the MR scan. The clinical features of the patients are depicted in Table 1.

**Table 1:** Clinical data of the patients.

	**Mean**	**SD**	**Min**	**Max**
Age	40.3	10.3	19.3	73.2
DD	9.82	5.29	0	28
EDSS	1.49	1.42	0	6.5
DMT (yes/no)	130/10			

*DD, disease duration; EDSS, expanded disability status scale; DMT, disease-modifying treatment*.

### Image Acquisition

All the MRI data were acquired on a 3T GE Discovery 750w scanner. We outlined the imaging protocol in our recent publication (17, 18). Structural images were acquired using 3D axial fast spoiled gradient echo (FSPGR) T1-weighted images (TR = 450 ms, TE = 4.2 ms, FOV = 256 mm, slice thickness 1 mm, flip angle 12), 2D spin-echo (SE) T1-weighted images (TR = 500 ms, TE = 4.2 s, FOV = 240 mm, slice thickness 1.4 mm, flip angle 73), 3D sagittal fluid-attenuated inversion recovery (FLAIR) (TR = 6.7 ms, TI = 1.8 ms, FOV = 250 ms, slice thickness 1.4 mm), and 3D double inversion recovery images (DIR) (TR = 7,000 ms, TE = 90 ms, TI = 546 ms, TI2 = 2,900 ms, FOV = 250 mm, slice thickness 1.4 mm), 2D axial T2 and proton density (PD) weighted dual echo fast spin echo sequences (TR = 3,000 ms, TE = Min Full, TE2 = 102 ms, FOV = 240 mm, flip angle 125, slice thickness 3.0 mm), and 2D coronal short tau inversion recovery (STIR) images on the optic nerve and the chiasm (TR = 3,000 ms, TE = 42 ms, TI = 185 ms, FOV = 240 mm, flip angle 111).

The ethics committee of the Medical University of Szeged approved the study and all study participants gave their written informed consent in accordance with the Declaration of Helsinki (Ref. No. 56/2011).

### Raters

Five raters who were qualified in the field of MS but are working in different specialties and on various levels: radiologist (IP: 14 years of experience in the field of MS), radiology resident (FK: 3 years of experience in the field of MS), neurologist (TK: 12 years of experience in the field of MS), neurology resident (ET: 4 years of experience in the field of MS), and a Ph.D. fellow (BB: working with MS for 2 years) were selected to evaluate the scans by lesion count and localization.

### Lesion Classification

The five raters evaluated MR images independently on identical workstations using the same report template. Images were evaluated on the Biotronics 3DNet PACS and visualization tool (Biotronics3D, London, UK).

The definition of a white matter lesion was ovoid T1/FLAIR hypo/hyperintensity larger than 3 mm. The following lesions were counted separately:

- Periventricular: ovoid T2 hyperintense lesions touching the lateral ventricles.
- Infratentorial: T2 hyperintense lesions in the brain stem and the cerebellum, an area extending from the tentorium to the level of the foramen magnum.
- Cortical/juxtacortical: supratentorial T2 hyperintense lesions in the cortex or touching the cortex.
- Optic nerve: T2 hyperintense lesions in the optic nerve between the bulbous and the optic chiasm.
- Spinal: T2 hyperintense lesions in the spinal cord between the level of the foramen magnum and the CIII-CIV intervertebral disc.

Periventricular lesions were evaluated on the sagittal FLAIR images. FLAIR and DIR images were used to identify the cortical and juxtacortical lesions. Coronal STIR images were used to evaluate the optic nerves. Infratentorial lesions were evaluated on the sagittal FLAIR images aided by the axial PD/T2 images. The FOV of the sagittal FLAIR and DIR images were selected in a way that made it possible to evaluate the first three cervical segments of the spinal cord. The total lesion count was calculated by adding the number of lesions in each location, excluding the optic nerve. The purpose of this variable was to see how the raters perform when they did not have to classify lesions by brain regions.

Atrophy was evaluated on axial 3D FSPGR images and measured by the rater's subjective opinion on a binary scale (significant or non-significant). Cases with a higher lesion number were allowed to have an error as seen in Table 2.

**Table 2:** Since counting the lesions is increasingly more difficult with a higher lesion count, we created arbitrary categories accounting for this inaccuracy.

**Category**	**0**	**1**	**2**	**3**	**4**	**5**	**6**	**7**	**8**	**9**	**10**	**11**
Lesion count	0	1	2	3	4	5–6	7–8	9–11	12–15	16–19	20–24	25+

*With this approach, readers had to estimate the lesion number above 5 lesions*.

### Statistics

For the statistical analysis, we used IBM SPSS software, a two-way mixed intraclass correlation (ICC) was calculated with an absolute agreement for single measures. The ICCs were primarily determined for all five raters and then retested, excluding the most divergent rater to see how much the results would improve.

To investigate the reproducibility of lesion detection in patients with low- and high-lesion counts, we divided the patients into two groups based on the median lesion count. We also repeated this analysis using only the four most accurate raters.

The results are interpreted by 95% CI. Categorization of the reproducibility was based on the following criteria (ICC): poor 0–0.5; moderate 0.5–0.75; good 0.75–0.9, and excellent 0.9–1 (19). Graphs were made by GraphPad Prism 8.0 software.

## Results

### Lesions by Regions

Averaging the lesion count by every rater, the results are shown in Table 3.

**Table 3:** Average lesion count per region.

	**Periventricular**	**Juxtacortical**	**Deep white** **matter**	**Infratentorial**	**Spinal**	**Black hole**
Mean	12.14	2.90	7.67	1.14	0.67	3.93
SD	10.016	4.246	9.955	1.508	0.849	4.511

### Reproducibility of Lesion Count, All Rater, All Patients

**Figure 1:**
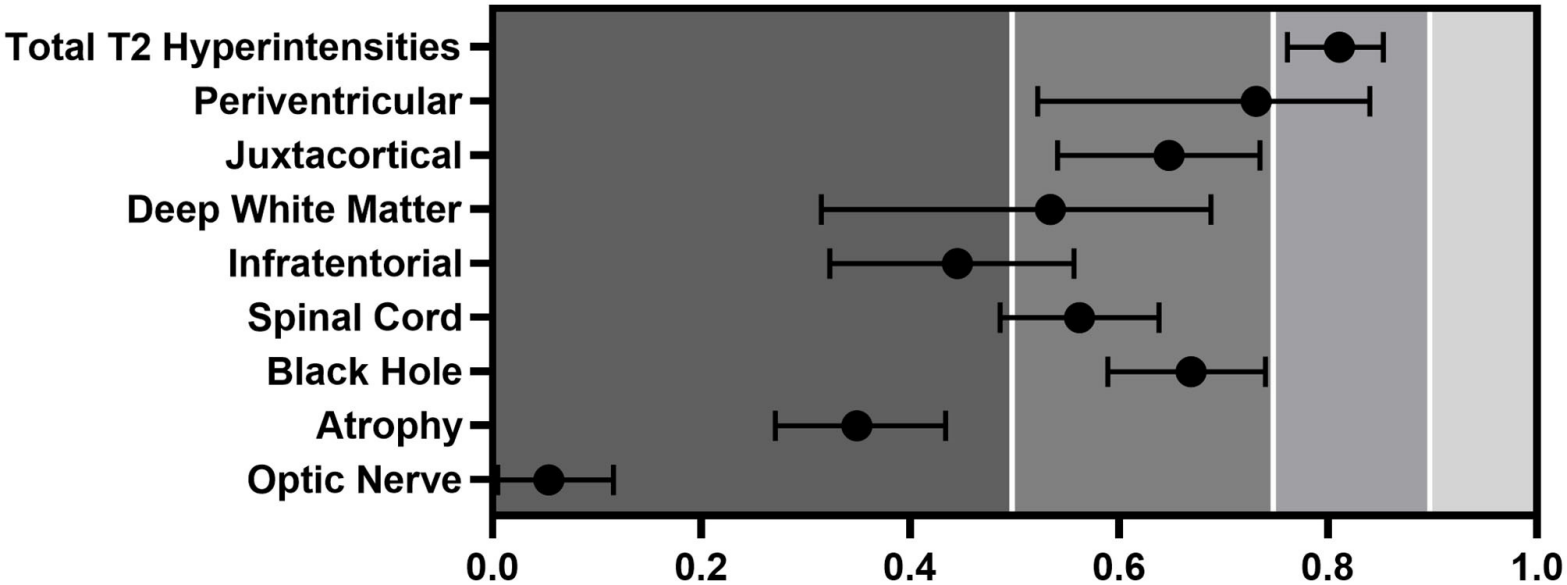
ICC of the different regions evaluated by all five raters. Error bars represent CIs.

The calculated ICCs for five raters were below the excellent (<0.90) reliability in every region (Figure 1; Table 4). The number of T2 hyperintense lesions in the cerebrum [periventricular, (juxta)cortical, deep white matter] had the best ICC (ICC: 0.761). The highest ICC was for the periventricular region (ICC: 0.731) which is considered a moderate–good result. Also, moderate results were given for juxtacortical lesions (ICC: 0.648) and for black holes (ICC: 0.669). Deep white matter (ICC: 0.534), infratentorial (ICC, 0.445), and spinal cord lesions (ICC: 0.562) had poor-to-moderate reproducibility. Poor reproducibility was noted for atrophy prediction (ICC: 0.349) and optic nerve involvement (ICC: 0.054).

**Table 4:** Summary of the ICCs and the 95% CIs made in this study.

**LC**	**Rater**		**Periventricular**	**Juxtacort**	**DWM**	**Infratentorial**	**Spinal**	**BH**	**Atrophy**	**Optic**	**Total T2** **HI**
All	5	CI	0.840	0.735	0.688	0.557	0.638	0.740	0.434	0.116	0.853
		ICC	**0.731**	**0.648**	**0.534**	**0.445**	**0.562**	**0.669**	**0.349**	**0.054**	**0.811**
		CI	0.522	0.541	0.315	0.323	0.486	0.589	0.271	0.005	0.761
	4	CI	0.893	0.815	0.749	0.606	0.704	0.778	0.456	0.202	0.909
		ICC	**0.846**	**0.762**	**0.603**	**0.527**	**0.634**	**0.71**	**0.364**	**0.118**	**0.882**
		CI	0.776	0.702	0.365	0.446	0.561	0.629	0.277	0.047	0.851
Low	5	CI	0.709	0.443	0.591			0.571			0.801
		ICC	**0.544**	**0.31**	**0.428**			**0.446**			**0.709**
		CI	0.314	0.196	0.240			0.326			0.591
	4	CI	0.800	0.546	0.660			0.639			0.852
		ICC	**0.716**	**0.406**	**0.499**			**0.51**			**0.788**
		CI	0.612	0.273	0.285			0.374			0.708
High	5	CI	0.614	0.693	0.542			0.614			0.684
		ICC	**0.421**	**0.556**	**0.337**			**0.474**			**0.581**
		CI	0.191	0.382	0.119			0.320			0.476
	4	CI	0.703	0.785	0.607			0.715			0.812
		ICC	**0.555**	**0.702**	**0.385**			**0.609**			**0.736**
		CI	0.356	0.603	0.114			0.492			0.647

*LC, lesion count; BH, black-hole; T2 HL, T2 hyperintense lesions. ICC values are highlighted*.

### Concordance Between Raters

**Figure 2:**
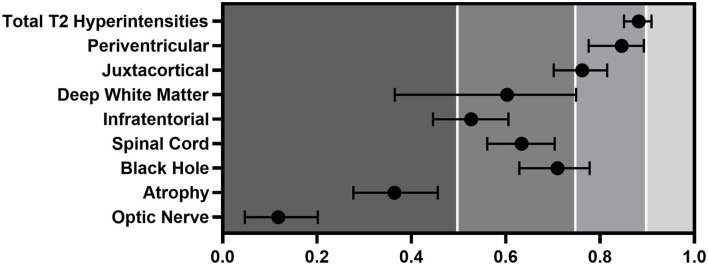
ICC with of the different regions evaluated by the four closest raters. Error bars represent CIs.

After leaving out the most outlier rater in every area, the ICCs improved between 0.015 and 0.115 (Figure 2). The most significant change and the best result was seen in the periventricular region, where we measured ICC to be 0.115 times higher than that with 5 raters, but it still did not reach the excellent level (ICC: 0.846). Juxtacortical (ICC, 0.762) and black hole (ICC: 0.710) values improved from moderate-to- good reliability. The spinal cord's ICC increased to moderate (ICC: 0.634) but infratentorial (ICC: 0.527) and deep white matter (ICC: 0.603) regions showed no significant quality change. Optic nerve (ICC: 0.118) and atrophy (ICC, 0.364) judgment showed the worst reliability even after leaving out the divergent rater. The sum of the lesions does reach the excellent range by a margin (ICC: 0.882).

**Figure 3:**
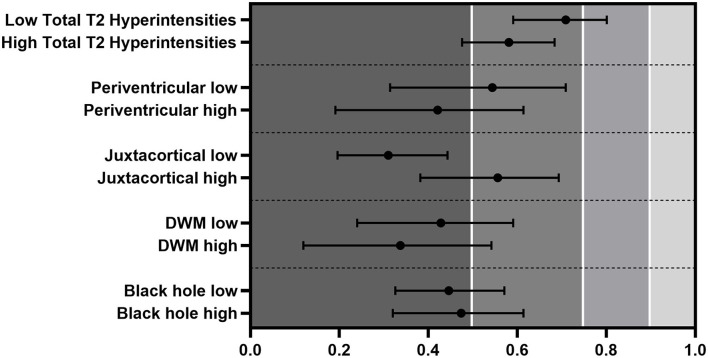
ICC differences between low- and high-lesion counts evaluated by every rater. Error bars represent CIs.

While in most of the regions, the ICCs improved marginally and the raters' performance did not differ significantly. By the leaving-one-out approach, the ICCs were very close (Figure 3).

### Reproducibility in High- and Low-Lesion Count Groups

**Figure 4:**
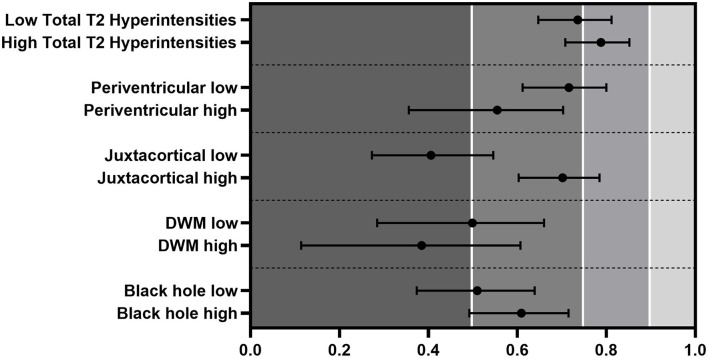
ICC differences between low- and high-lesion counts evaluated by the four closest. The classification of juxtacortical lesions was significantly lower when the juxtacortical lesion burden was low. Error bars represent CIs.

We divided the patients into low- and high-lesion count groups based on the median lesion count of all five raters. ICCs were in the poor-to-moderate range, except for the sum of all lesions that reached moderate-to-good quality in the low-lesion count subjects (low ICC: 0.709 vs. high ICC: 0.581) (Figure 4). Periventricular [low (ICC: 0.544) vs. high (ICC: 0.421)] and DWM [low (ICC: 0.428) vs. high (ICC: 0.337)] regions showed better ICC on the lower side, and black holes presented minimal changes (low ICC: 0.446 vs. high ICC: 0.474). Interestingly, the lesion identification in the juxtacortical area performed much better, although not significantly in the higher lesion load group (low ICC: 0.310 vs. high ICC: 0.556).

The same analysis was performed using only the four best raters. The quality of the ICCs improved similarly to what we have seen with five raters (Figure 4). In the juxtacortical region, patients with a high-lesion load showed significantly higher ICC than the low group (low ICC: 0.406 vs. high ICC: 0.702). Black holes presented a slightly greater ICC in the high group (low ICC: 0.510 vs. high ICC: 0.609). Periventricular (low ICC: 0.716 vs. high ICC: 0.555) and DWM (low ICC: 0.499 vs. high ICC: 0.385) areas showed better ICC between raters in patients with a lower lesion count, but not significantly. The quality of the tests mostly stayed in the moderate-to-good range.

## Discussion

In this study, we measured interrater reliability by lesion number and localization in patients with multiple sclerosis. Overall, the intraclass correlation coefficients showed high variability between different areas, although they never reached the excellent range.

**Figure 5:**
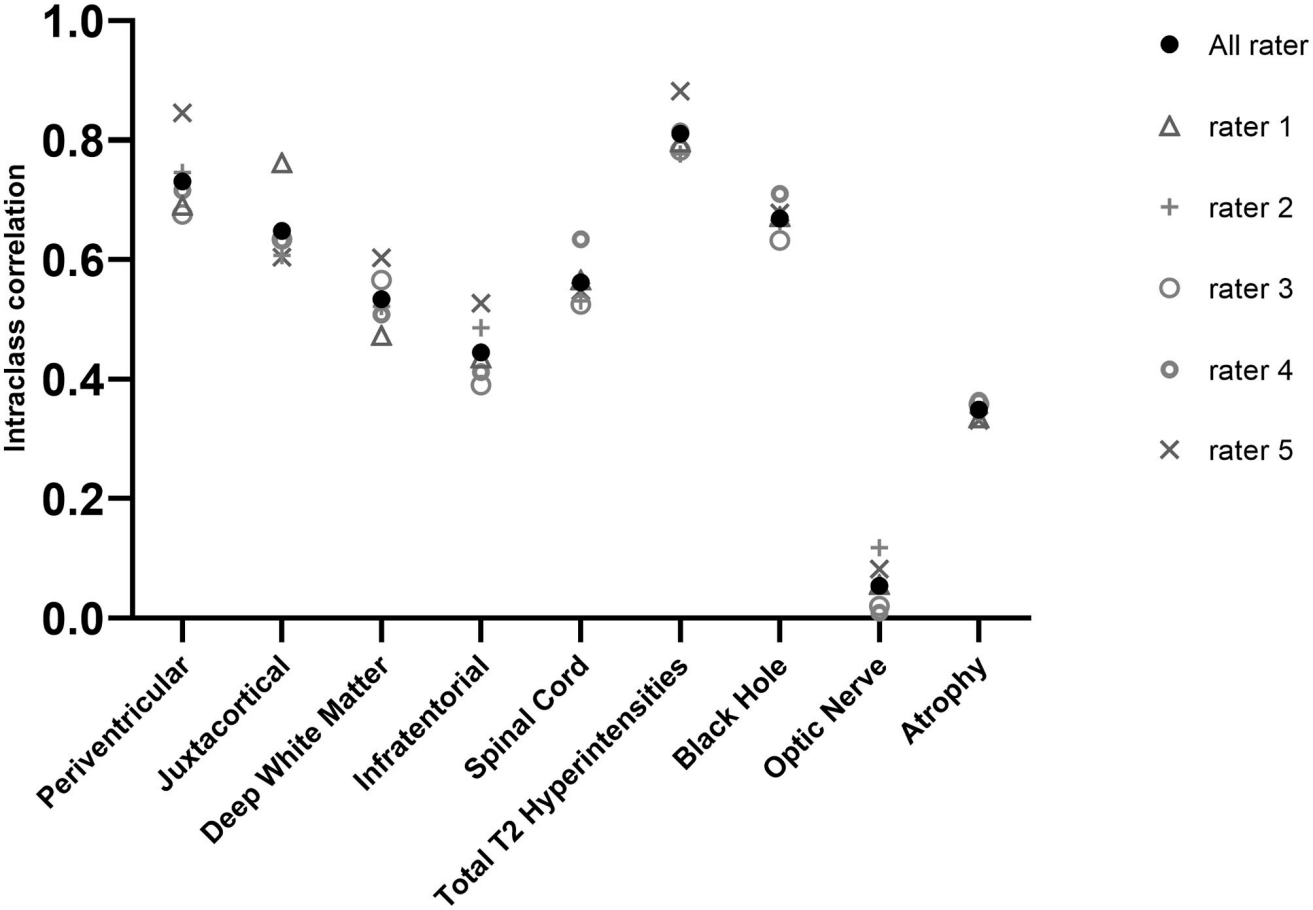
ICCs for all raters and when we left out different ones. For example, *rater 1* shows ICC for the other 4 thus a higher value means lower performance for the rater.

Unexpectedly, abandoning the most divergent rater could not improve the ICC into the excellent quality range, even if a considerable improvement was seen in some regions. It was not possible to discriminate between the raters by experience, as their performance was close to each other (Figure 5), which means that they performed consistently throughout the study. The sum of the lesions having the highest ICC showed that the overall lesion count is reliable, but classifying them by regions is a difficult task.

Grouping by lesion numbers with the four best raters showed us that the juxtacortical lesions' ICC in the high-lesion load group was significantly higher than in the low-lesion group, seeing a tendency for this with black holes also. These results suggest that lesions in brain regions with little lesion probability (Table 3) have a higher chance for detection if the lesion count in that area is larger. The simple explanation for this can be that even a small number of lesions in a low-probability region is easier to detect than one or two lesions. We observed the opposite in regions where lesion number is normally high (periventricular, DWM); the ICC was higher if the lesion count was lower. The reason behind this could be similar, that is, the raters have an easier task to keep the counting on track with fewer lesions. Also, high-lesion load can be confluent, which is challenging to work with.

The explanation for this could be that these lesions are hard to detect even for experts using DIR sequences (20), while if a patient has a higher lesion number, then maybe they can no longer keep count and are more easily missed. The opposite of this was presented when we got higher ICC for low-lesion load in regions where the lesions are more common (periventricular, DWM), which can be explained by the difficulty of counting, as they could be confluent.

Altogether, it seems that the lesions could be best evaluated if their number is in the mid range. If it is too low, they could be missed easier and if it is too high then counting is more challenging with the appearance of the confluent inflammation.

The area in which new lesions appear is especially critical. In recent years, plenty of studies found evidence of clinical and cognitive impairment in association with cortical lesion load (7, 21, 22). Detecting these lesions could be challenging on FLAIR images, and though the DIR scan shows higher sensitivity (23), this sequence is not yet part of the conventional MS MRI protocol.

There is no consensus on whether optic nerve involvement should be in the diagnostic criteria. The 2016 MAGNIMS guideline (1) used optic lesions as a diagnostic marker but the 2017 revised McDonald criteria (2) excluded them, due to missing validation and it suggested further research to be conducted. We showed that optical lesions are difficult to spot and the ICC was lower than 0.2, which can be considered as a random classification that supports the view that diagnosis of MS should not be based on optic nerve findings.

Despite the results, managing a patient with MS can undoubtedly be aided with the quality of the report. Studies showed that structured MRI reports that use a template for a broad and proper description of MS findings contained significantly more adequate information relevant to MS clinical decision-making than non-structured reports (11, 12).

Lesion segmentation tools are available with different methodological approaches. Semiautomated software including threshold-based, fuzzy connectedness or seed-based region growing methods are still time-consuming and have limitations with large data (24). Automated segmentation algorithms become popular recently because of their high performance (25–30). The disadvantage of these approaches (e.g., the supervised algorithms) is the need for thorough training. Unsupervised methods do not have these limitations, albeit their results might be less accurate as they do not have any prior input data to train from (23).

The demand for reliable lesion segmentation software is growing. In the international MICCAI challenge (24) in 2016, 13 teams contested against the grand truth set by a record number of seven experts. The results showed that even the best method was by far outperformed by the experts. The study also showed a positive correlation between lesion load and volume with the three measured parameters, which means performance was worse when a patient had a smaller lesion number or volume. Automated approaches also had serious problems with data from a new unknown scanner. These data call attention to how important it is to know the exact performance of human readers in lesion detection, as some of the automated approaches are trained on these data.

To the best of our knowledge, this is the first study that investigates the interrater reliability of the lesion classification in different areas using a number of raters. Although there were some studies in which the interrater reliability was measured, but only for diagnostics. In the first study (14), the interobserver agreement between four neurologists was tested for diagnosing MS by the McDonald's and Poser's criteria. The results showed moderate-to-substantial agreement. In the second study (15), the interobserver agreement on the radiological criteria of the International Panel was investigated where four trained and four inexperienced radiologists were compared. The IP-trained radiologists reached moderate-to-substantial agreement and excellent agreement on the enhancing lesions while the non-trained group performed notably poorly.

Our study also has some limitations. First and most importantly, we did not manage to recruit five neuroradiology experts which could have made our results closer to reality. Despite our raters working in different fields, they all have the necessary experience with MS to make this study valid. Second, a grand truth was not established thus we cannot determine how much the raters deviated from it, but we can only see how close they were to each other. Third, intrarater correlation could have added an extra value to the study, but the limited resources did not make it possible to evaluate the scans for a second or third time. Finally, optic nerve hyperintensities were only examined on STIR sequences but not on FLAIR or DIR images, which could have improved our detection quality.

In summary, our study indicates that finding new lesions or diagnosing the disease can be overlooked even by experts that can influence disease outcomes. With the rapid development of artificial intelligence, the use of automated lesion segmentation software will eventually emerge into the clinical routine and will be a useful tool for (reporting) radiologists.

## Data Availability Statement

The raw data supporting the conclusions of this article will be made available by the authors, without undue reservation.

## Ethics Statement

The studies involving human participants were reviewed and approved by University of Szeged (Ref. No. 56/2011). The patients/participants provided their written informed consent to participate in this study.

## Author Contributions

Guarantor of integrity of the entire study and manuscript editing: ZK. Study concepts and design: KB and PK. Literature research, statistical analysis, and manuscript preparation: BB. Experimental studies/data analysis: ET, IP, FK, NS, and BB. All authors contributed to the article and approved the submitted version.

## Conflict of Interest

The authors declare that the research was conducted in the absence of any commercial or financial relationships that could be construed as a potential conflict of interest.

## Publisher's Note

All claims expressed in this article are solely those of the authors and do not necessarily represent those of their affiliated organizations, or those of the publisher, the editors and the reviewers. Any product that may be evaluated in this article, or claim that may be made by its manufacturer, is not guaranteed or endorsed by the publisher.
